# Multifunctional electrospun asymmetric wettable membrane containing black phosphorus/Rg1 for enhancing infected wound healing

**DOI:** 10.1002/btm2.10274

**Published:** 2021-12-15

**Authors:** Liming Zhou, Nanbo Liu, Longbao Feng, Mingyi Zhao, Peng Wu, Yunfei Chai, Jian Liu, Ping Zhu, Rui Guo

**Affiliations:** ^1^ Key Laboratory of Biomaterials of Guangdong Higher Education Institutes, Guangdong Provincial Engineering and Technological Research Centre for Drug Carrier Development, Department of Biomedical Engineering Jinan University Guangzhou China; ^2^ Guangdong Cardiovascular Institute, Guangdong Provincial People's Hospital Guangdong Academy of Medical Sciences Guangzhou Guangdong China

**Keywords:** asymmetric wettable membrane, black phosphorus, electrospinning, ginsenoside Rg1, wound infection

## Abstract

Bacterial infection is one of the most frequent complications in the burn and chronic wounds. Inspired by natural existing superhydrophobic surface structures, a novel asymmetric wettable membrane was prepared using the electrospinning technique for facilitating the bacteria‐infected wound healing. Herein, the prepared membrane consists of two layers: The hydrophobic outer layer was composed of poly (lactic‐co‐glycolic) acid (PLGA) and black phosphorus‐grafted chitosan (HACC‐BP), while the hydrophilic inner layer was composed by using a mixture of gelatin (Gel) with ginsenoside Rg1 (Rg1). Biological studies in vitro showed BP@PLGA/Gel (BP@BM) membrane with excellent antibacterial activity could significantly inhibit the adhesion of bacteria, and Rg1 could facilitate the migration and tube formation of human umbilical vein endothelial cells (HUVECs). Compared to Aquacel Ag dressing, the result in vivo revealed that the Rg1/BP@BM could facilitate better wound healing by triggering phosphoinositide 3‐kinase (P‐PI3K/PI3K) and phosphorylation of protein kinase B (P‐AKT/AKT) signaling pathways, upregulating Ki67, CD31, α‐SMA, and TGF‐β1, and downregulating TNF‐α, IL‐1β, and IL‐6, promoting M2 polarization (IL‐10, CD206, and Arg‐1) of macrophages, inhibiting M1 polarization (iNOS) of macrophages. These findings suggested that the asymmetric wettable membrane have the huge potential for wound healing.

## 1 INTRODUCTION

Bacterial infection of a skin wound was the main reason of delayed wound healing, and high bacterial levels will hinder the progression of wound healing.^1^ Unfortunately, prior study reported that infection caused by antimicrobial‐resistant pathogens may lead to 10 million annual deaths by the year 2050.^2^ Bacterial infection was usually accompanied by inflammation.^3^ Persistent inflammation will eventually cause cell death and tissue necrosis, leading to inflammation or injures of surrounding soft tissues.^4^ Furthermore, excessive inflammation response was one of the significant reasons for delayed wound healing, which may trigger severe complications.^5^ Different wound dressings such as gauze, foam, and bandages have been presented to obtain the temporary barriers.^6^, ^7^ However, most of them were not compatible with the compatible environment to completely remodel cell layer and functions of skin.^8^, ^9^ Therefore, there is an urgent need for developing an ideal wound dressing in chronic infected wounds.

Asymmetric membrane was an emerging functional dressing since they can mimic construction of skin and furnish the compatible environment for wound healing.^10^ Asymmetric membranes have a compact outer layer which similar to skin epidermis, exhibit hydrophobicity that could inhibit the adhesion of the bacteria and prevent the loss of water.^1^ Moreover, the hydrophilic inner layer played the role of skin dermis, which could provide an ideal microenvironment of wound healing, and further promote delivery of nutrition and remove the excess exudates.^11^ Owing to their extraordinary properties containing water retention, high mechanical properties, and biocompatibility, asymmetric membranes have been applied in the biomedical field such as wound healing^12^ and sensor.^13^

As a novel and grow rapidly technique, electrospinning has attracted tremendous attention of the biomaterial field owing to its simplification, multifunction, and high efficiency.^8^ Besides, electrospun membranes have an ideal loading capacity of drug owing to the high surface area.^14^ Many kinds of polymers such as polyurethane (PU),^15^ polyacrylonitrile (PAN)^16^ and poly(ethylenimine) (PEI),^17^ have been applied to produce the asymmetric membranes by electrospinning. However, some polymers like PU, having completely dense structure which made the membranes with low water vapor transmission rate (WVTR), may cause the accumulation of exudate and complications of infection‐related in clinic.^18^ Poly (lactic‐co‐glycolic) acid (PLGA), due to its biodegradability and biocompatibility properties, has been approved applications in therapeutic devices by the Food and Drug Administration.^19^ Besides, it has adjustable water vapor permeability^20^ and excellent mechanical properties,^21^ making it has great potential to be applied in wound dressing.

Black phosphorus (BP), a rising two‐dimensional (2D) nanomaterial, could kill the bacteria under the near‐infrared ray (NIR) and cause irreversible damage through high photothermal conversion efficiency.^22^ BP had huge potential in the field of biomaterials because of its remarkable biocompatibility. Phosphorus was inherent elements in the human body,^23^ and the degradation products of BP were phosphates and phosphonates, which were harmless to human.^24^ Furthermore, ginsenoside Rg1 (Rg1) was one of the protopanaxtriol‐type saponin, which could be extracted from Traditional Chinese Medicine Panax ginseng.^25^ Previous studies reported that the Rg1 had abundant pharmacological activities such as cardiovascular protection, anti‐oxidative and anti‐inflammation,^26^ and also made progress in the treatment of diabetes,^25^ nervous system diseases^27^ and cardiovascular diseases.^26^ Unfortunately, to the best of our knowledge, asymmetric membranes loading BP and Rg1 to facilitate the infected wound healing has not been reported.

**SCHEME 1:**
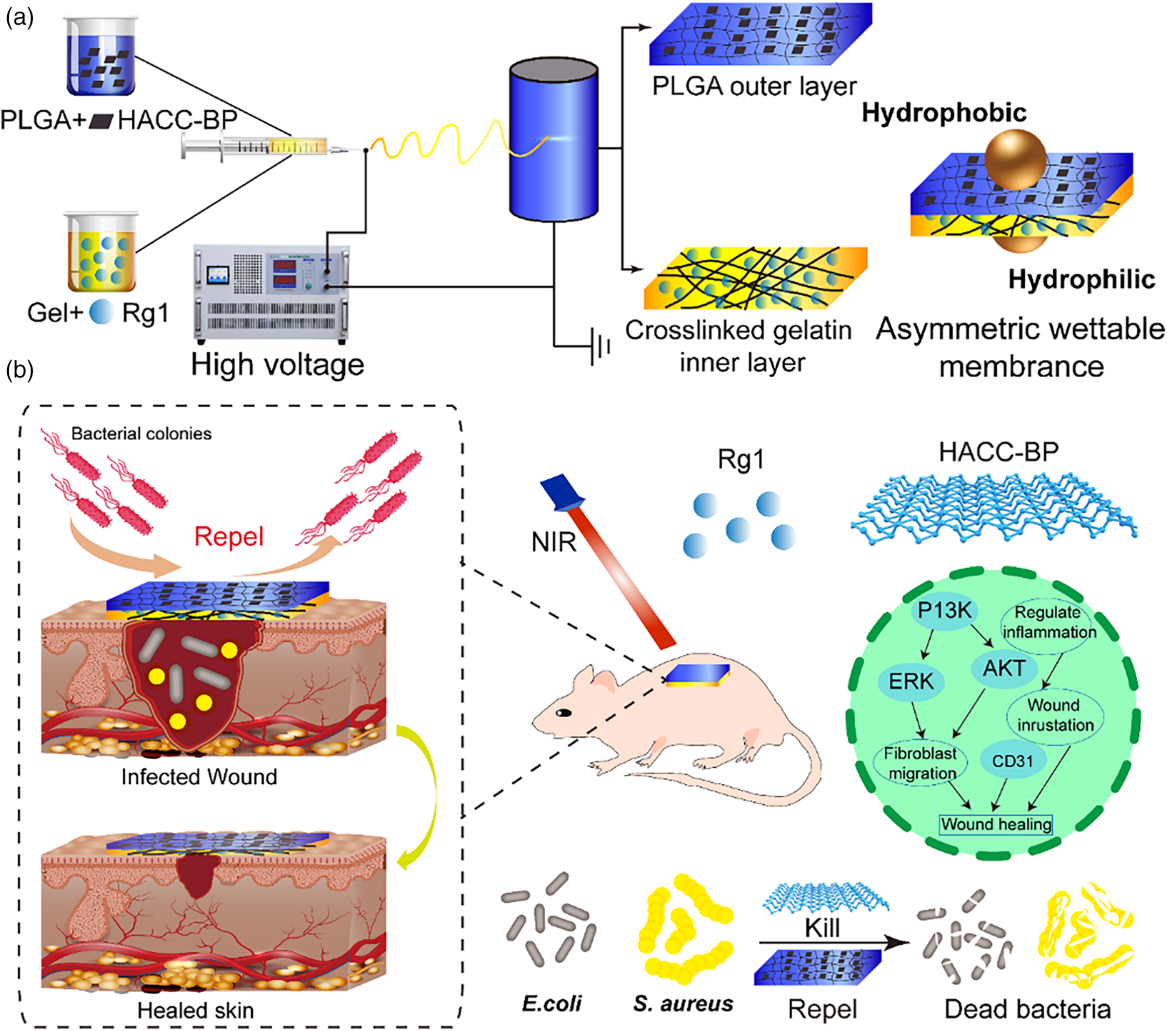
(A) Schematic of the preparation of the asymmetric wettable membrane. (B) The asymmetric wettable membrane can prevent and kill bacteria and allows controlled Rg1 release to promote the bacteria‐infected wound healing

Therefore, we prepared the electrospinning asymmetric wettable membrane consisting of outer layer and inner layer (Scheme 1). The first layer was composed of PLGA and BP graft chitosan (HACC‐BP), while the second layer was composed by using a mixture of gelatin (Gel) with Rg1. The prepared asymmetric wettable membrane exhibited excellent mechanical properties and suitable moisture, which was not only available for cell proliferation and water vapor permeation, but also had a good antibacterial effect. Antibacterial activity in vitro showed that BP@PLGA/Gel (BP@BM) hydrophobic outer layer could prevent bacterial colonization, and had good antibacterial activity. Besides, cytotoxicity in vitro revealed that Rg1 could facilitate the migration and tube formation of HUVECs. Compared to other groups, the result in vivo revealed that the Rg1/BP@BM group could regulate growth factors, inflammatory factors and chemokines, inhibited inflammation, promote collagen deposition, cell proliferation, and angiogenesis, which further confirmed the potential value of Rg1/BP@BM for wound dressing and other biomaterials flied.

## 2 EXPERIMENTAL SECTION

### 2.1 Materials and reagents

Poly (lactic‐co‐glycolic) acid (PLGA, Mw = 17 kDa) was purchased from Daigang Bio Engineering Co., Ltd. (Jinan, China). Gelatin (Gel, type A, from porcine) and dimethyl sulfoxide were purchased from Sigma‐Aldrich (Shanghai, China). Ginsenoside Rg1 and hexafluoroisopropanol were purchased from Aladdin Industrial Corporation (Shanghai, China). Quaternized chitosan (HACC, 90% degree of substitution) was obtained from Lushen Biological Technology Co., Ltd. (Nantong China). Dulbecco's modified Eagle's medium (DMEM), fetal bovine serum (FBS), and penicillin/streptomycin were purchased from Gibco (GIBCO, USA). Counting Kit‐8 (CCK‐8) was purchased from BestBio Bio‐Technology Co., Ltd. (Shanghai, China).

### 2.2 Preparation of the membrane

#### 2.2.1 Preparation of hydrophobic PLGA layer

PLGA was completely dissolved in hexafluoro isopropanol to obtain a 10% (w/v) PLGA concentration by constant continuous stirring for 12 h. The solution was then filled into a 20 ml syringe using the 22# needle (inner diameter of 0.4 mm) and controlled by a syringe pump at a flowing rate of 2.5 ml/h at a voltage of 15 kV. The needle tip to collector distance was 12 cm. After electrospinning, the resulting electrospun membranes (a thickness of 0.15 mm) were stored in a desiccator.

#### 2.2.2 Preparation of hydrophilic gel layer

Gel (10% (w/v)) was dissolved in 10 ml hexafluoro isopropanol under continuous stirring for 6 h at 60°C to obtain an electrospinning solution of the hydrophobic layer. The parameters of electrospinning were: A flowing rate of 2.5 ml/h, a voltage of 15 kV, and the needle tip to collector distance was 12 cm. Afterward, the Gel membrane was treated with 75% ethyl alcohol solution containing 50 mM EDC and 25 mM NHS for 24 h as crosslinking step. Finally, the obtained Gel membrane was washed with 75% ethyl alcohol to remove any residual EDC/NHS, and then dried under vacuum at 25°C. Finally, the resulting crosslinked Gel membrane (a thickness of 0.15 mm) were stored in a desiccator.

#### 2.2.3 Preparation of asymmetric wettable membrane

The asymmetric wettable membrane was prepared by continuous electrospinning technique. Briefly, PLGA nanofiber membrane was obtained by electrospinning, and the Gel or Gel/Rg1 was spun continually and covered onto the surface of the PLGA nanofiber membrane, which could obtain an uncrosslinked asymmetric wettable membrane. Then, the above membrane was crosslinked by the EDC/NHS method. Finally, the crosslinked asymmetric wettable membrane could be obtained after drying, named as BM.

### 2.3 Characterization of the membrane

#### 2.3.1 SEM, TEM, and DLS characterization

The surface micromorphology of the electrospinning asymmetric membranes was observed via a field emission scanning electron microscope (Carl Zeiss EVO LS 10, Oberkochen, Germany). The nanofiber diameters were calculated by the ImageJ software. Transmission Electron Microscopy (TEM) images were captured by transmission electron microscopy (JEM 2100F, JEOL, Japan) using a copper grid. Hydrodynamic diameter of HACC‐BP was characterized by Zetasizer nanoZS instrument (Nano‐ZS; Malvern Instruments, UK).

#### 2.3.2 Water contact angle measurement

The hydrophobic layer and the hydrophilic layer were dried overnight. Briefly, a volume of about 5 μl of distilled water was dispensed from the syringe onto the membrane's surface. After 5 s, the images of water droplets on the membrane's surface were captured by a water contact angle instrument (DSA25; KRUSS, Germany), and water contact angle of each membrane was calculated.

#### 2.3.3 Water vapor transmission rate

The WVTR of the asymmetric membrane was measured by the standard method described in the American Society for Testing and Materials (ASTM) E96‐00.^28^ In brief, the membranes were cut into a suitable size, and then mounted on the mouth (diameter 13 mm) of glass bottles containing 10 ml of deionized water. Then the membrane was transferred to a constant temperature and humidity incubator (temperature set 37°C, relative humidity was maintained at 79%). A glass bottle without membranes was used as a blank control. The weight of the sample was recorded at predetermined time points. The WVTR is calculated according to the following formula:

$$\text{WVTR}=\frac{\Delta m/\Delta t}{A.}$$

where Δ*m*/Δ*t* represents the weight of water loss in 24 h (g/day) and A represents the surface area of the bottle mouth (m^2^).

#### 2.3.4 Mechanical properties

The mechanical properties of the materials are measured according to the test method of the pharmaceutical industry standard YY/T 0471.4‐2004.^29^ The tensile strength and Young's modulus were measured by an universal tester (ELF3200, U.S.) with a stretching rate of 6 mm/min. Rectangular specimens of each membrane (1 cm in width × 7 cm length) were stretched.

#### 2.3.5 In vitro swelling behavior and degradation behavior

The electrospinning membrane was cut into a 20 mm × 20 mm square. The Gel, PLGA, and PLGA/Gel were immersed in PBS solution (pH = 7.4) to assess the swelling behavior, and in PBS with 0.02 U/ml collagenase to evaluate the degradation behavior at 37°C, respectively. The swelling ratio (SR) was calculated according to the following formula:

$$\mathrm{SR}\;\left(\%\right)=\left(M_{t}–M_{0}\right)/M_{0}\times 100\%$$

where M_0_ represents the initial weight and M_t_ is the weight at predetermined time points of the membranes, respectively.

The Gel, PLGA, and PLGA/Gel membranes (20 mm × 20 mm) were placed in PBS containing 0.02 U/ml of collagenase at 37°C and 100 rpm. At predetermined time intervals, the membranes were collected, lyophilized, and weighed. The degradation ratio was calculated according to the following formula

$$\text{degradation ratio}\;\left(\%\right)=W_{t}/W_{0}\times 100$$

where W_0_ represents the initial weight of the membranes and W_t_ is the weight of the degraded membranes at predetermined time points, respectively.

#### 2.3.6 In vitro drug release of Rg1

The drug release of Rg1 was assessed as follows: The dried membrane with a mean weight of 0.1 g was immersed in 10 ml of PBS (pH = 7.4) at 37°C and irradiated under an 808 nm NIR laser for 5 min. Subsequently, 1 ml of incubation liquor was taken out at predetermined time points, and an equal volume of fresh medium was replenished. The released amount of Rg1 was detected by UV spectrophotometry at 203 nm.

### 2.4 Photothermal performance of the membranes

Prepared electrospinning membranes with different concentrations of BP (150, 300, and 500 μg/ml). The electrospinning membranes were placed in a 24‐well plate and irradiated under an 808 nm NIR laser for 5 min with laser densities of 1.5 W/cm^2^, recording temperature every 10 s. The thermal images were captured with a thermal imaging camera (Fotric 222, Shanghai, China).

### 2.5 In vitro antibacterial property

To evaluate the photothermal antibacterial properties of membranes in vitro, *Escherichia coli* and *Staphylococcu*s *aureus* and *Pseudomonas aeruginosa* were used as model bacteria. The membranes were placed into a 24‐well plate. Then, 1.5 ml bacterial suspension (1 × 10^6^ CFU/ml) was dropped in the plate and irradiated with an NIR laser (808 nm; laser density 1.5 W/cm^2^) for 5 min. After 16 h of incubation at 37°C, the antibacterial behavior of each test sample was also studied by agar plate counting. Meanwhile, the OD values of the suspension were obtained by a microplate reader (IDE2000, COOC, China) at 600 nm.

The bacterial SEM was used to visualize the changes of cell morphologies with different materials under different conditions. The above bacterial liquid was centrifuged at 5000 rpm for 20 min to collect bacterial precipitation. The collected bacterial precipitation was fixed with 2.5% glutaraldehyde for 3 h, then the glutaraldehyde was removed by centrifugation at 5000 rpm for 20 min, and the fixed bacteria were washed twice with PBS and twice with deionized water. Finally, the treated bacterial suspension droplets were dropped on the single‐crystal silicon, and then observed by the SEM (EVO MA 15/LS 15, Carl Zeiss, Germany).

### 2.6 Cytocompatibility of the membranes

To evaluate the biocompatibility of the membranes, the mouse fibroblasts (3T3 cells) and human umbilical vein endothelial cells (HUVECs) were used. Cells were cultured in DMEM medium supplemented with 10% of FBS and 1% of penicillin–streptomycin in 5% CO_2_ at 37°C. Cells were harvested at ~80% confluence and then shifted to DMEM medium. Cells were seeded into 24‐well tissue culture plates at a density of 2 × 10^4^ cells/ml in each well. Afterward, the membranes were added and co‐cultured for different times (1, 2, and 3 days). The CCK‐8 would be used to assay the cytotoxicity and cell proliferation. For the CCK‐8 assay, each well was added with 500 μl of CCK‐8 reagent and incubated for 30–60 min at 37°C. Then transferred 100 μl to per well of the 96‐well plate, the OD value was determined using a microplate reader (IDE2000, COOC, China) at 450 nm. The Live/Dead staining was used to observe the live (green) or dead (red) state of cell after co‐culture with the membranes. For the Live/Dead assay, each well was added with 400 μl of staining regent. After 15 min of incubation at 4°C in the opaque background, the live cells could be observed at 490 nm while dead cells at 545 nm by inverted fluorescence microscope (Nikon TE2000‐S, Tokyo, Japan).

### 2.7 Scratch wound assay

The effects of asymmetric wettable membranes on HUVECs migration were evaluated with a wound healing scratch assay. HUVECs cells were seeded into 24‐well plates and grown until forming confluent monolayer. Subsequently, the cell monolayer was gently scratched in a straight line using a tip of sterile pipette, and then washed in PBS to remove residual cell debris. Afterward, to the artificial wound was added 500 μl of serum‐free medium and then a sterilized membrane was added. At predetermined time points, the bright field images of cells were taken by a microscope, and the cell migration rate was calculated according to the following formula:

$$\text{Cell migration}\;\left(\%\right)=\frac{A_{0}-A_{t}}{A_{0}}$$

where *A*_0_ represents the scratch wound area at 0 h and *A*_t_ is the wounded area measured at each time point after scratching.

### 2.8 Cell attachment study

The membranes were put into a 48‐well plate and soaked in 2 ml PBS to reach a saturated state. Afterward, the 3T3 and HUVECs cells were seeded on the membranes at a density of 2 × 10^4^ cells/ml and incubated for 48 h. Then, the samples were washed with PBS three times and fixed in 2.5% glutaraldehyde for 4 h, and then rinsed with PBS three times for 15 min. Finally, the obtained samples were dehydrated with graded concentration of ethanol (30%, 50%, 70%, 85%, 90%), and kept in a vacuum oven. The growth status of cells on the membranes were taken on scanning electron microscope.

### 2.9 Angiogenesis test

Matrigel and 96‐well plates were thawed at 4°C overnight. Afterward, 50 μl of Matrigel was dropped into a precooled 96‐well plates, and then placed statically at 4°C for 5 min. Subsequently, 100 μl HUVECs were seeded on each well at a density of 1 × 10^4^ cells/ml, and the FBS‐free medium containing different membranes were added. After incubation for 4, 6, and 12 h, the angiogenic capacity of membranes on HUVECs was recorded by a microscope.

### 2.10 In vivo wound healing experiments

All animal experimental protocols have been reviewed and approved by the Animal Protection and Use Committee of Jinan University. Sprague–Dawley rats were anesthetized with 3% sodium pentobarbital (60 mg/kg) via intraperitoneal injection. The backs of the rats were shaved and disinfected using 75% alcohol. Four standardized full‐thickness skin wounds (12 mm in diameter) were created on each rat by excising the dorsum. Afterward, 100 μl of mixed bacterial suspension (10^8^ CFU/ml) of *S. aureus* and *E. coli* would be uniformly spread on the wound, and 3 h later were covered with gauze, BM, Rg1@BM, BP@BM, Rg1/BP@BM or Aquacel Ag, respectively. Then, the membranes were irradiated by NIR laser (808 nm, 1.5 W/cm^2^) for 5 min. The gauze represented the negative control group, whereas the Aquacel Ag dressing (ConvaTec, Berkshire, England) represented the positive group. The wound was photographed at predetermined time points (0, 3, 7, and 14 days) and sacrificed at fixed time points.

### 2.11 Number of bacteria per wound

The excising skin tissues were collected on day 3, homogenized in 2 ml of sterile 0.9% saline solution. Then, the above solutions were plated on VRBA‐MUG and Mannitol salt agar selection medium agar plates. VRBA‐MUG were used for the selection of *E. coli* (white) and Mannitol salt agar selection medium was used for the selection of *S. aureus* (yellow gold colony), respectively.

### 2.12 Histology and western blot analysis

Tissue samples were firstly fixed with 4% paraformaldehyde and embedded into paraffin. Four‐μm sections were stained with H&E and collagen was stained with Masson's trichrome. For immunohistochemistry and immunofluorescence, sections were incubated overnight with primary antibodies to CD31, α‐SMA, IL‐10, CD206, Arg‐1, TGF‐β, TNF‐α, and IL‐1β. All other steps of immunohistochemistry and immunofluorescence staining were carried out according to manufacturer's protocol. The samples were photographed under a fluorescence microscope (Zeiss, Axio Observer D1, Germany). For WB analysis, skin samples were completely homogenized with lysis buffer, and tissue debris was removed by centrifugation. Proteins were separated on 10% sodium dodecyl sulfate (SDS)‐polyacrylamide gels. The protein level of Ki67, CD31, α‐SMA, P‐PI3K/PI3K, P‐AKT/AKT, PERK/ERK, TGF‐β1, TNF‐α, IL‐1β, and GAPDH of supernatant of each sample was investigated by the western blot (WB) method.

### 2.13 Statistical analysis

Quantitative data sets were performed as mean ± standard deviation (SD). Differences among the groups were assessed by the software SPSS (version on 19.0) using one‐way analysis of variance (ANOVA). *p < 0.05, **p < 0.01, and ***p < 0.001 were considered statistically significant.

## 3 RESULTS AND DISCUSSION

### 3.1 Characterization of the asymmetric membrane

**FIGURE 1:**
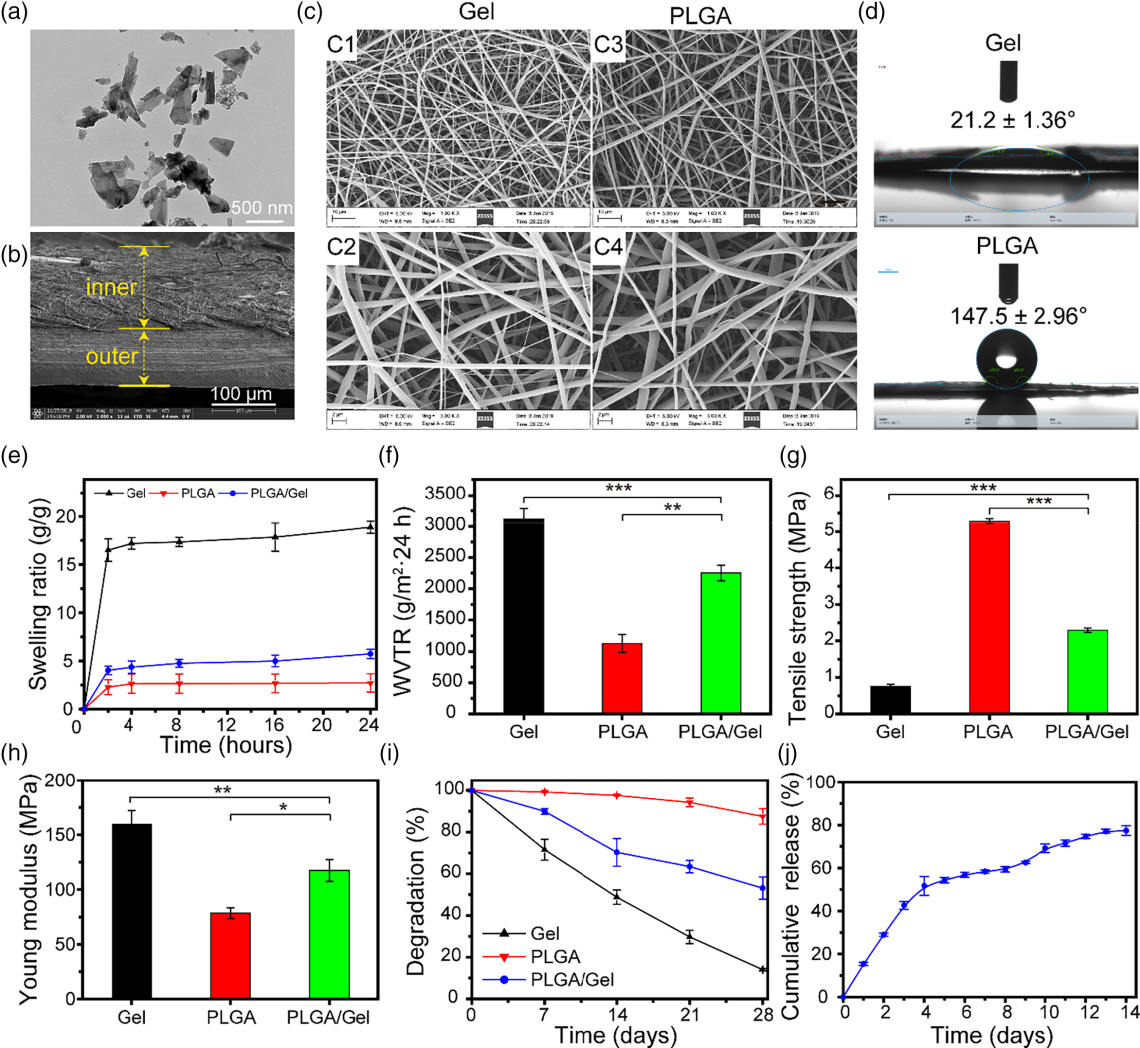
Characterization of the asymmetric membrane. (A) TEM image of HACC‐BP. (B) SEM cross‐section image of asymmetric membrane. (C) SEM image of the Gel layer and PLGA layer. (D) Water contact angles images of the hydrophilic surface of Gel layer and hydrophobic surface of PLGA layer. (E) In vitro swelling behavior of membrane immersed in PBS buffer at 37°C. (F) WVTR of different materials. Tensile strength and (G) Young's modulus (H) of Gel, PLGA, and Gel/PLGA. (I) In vitro degradation behavior of membrane. (J) In vitro cumulative release curve of Rg1

The TEM image showed that the HACC‐BP have a uniform 2D sheet‐like morphology (Figure 1A). The DLS showed that the diameter of HACC‐BP was at the range about 238 ± 5.6 nm (Figure S1). As shown in Figure 1B, the developed asymmetric membrane consisted of bilayer structure that could be easily distinguished. The optical images of the asymmetric membrane are exhibited in the Figure S2. As shown in Figure 1C, the cross‐section of SEM showed the layer presented the remarkably uniform and smooth nanofiber structure, which exhibited the characteristics of porous three‐dimensional, bead‐free, and branch‐free. Meanwhile, the nanofiber diameters of Gel and PLGA nanofibers were about 500–1000 nm. Besides, the water contact angle can be utilized to assess hydrophilic and hydrophobic behaviors of the gel layer and PLGA layer. The surface of solid represented hydrophilic when the contact angle assessed below 90°, while the solid surface exhibited hydrophobic if the contact angle exceeded 90°.^30^ In addition, the preparation of hydrophobic layer is inspired by the “lotus leaf effect.” As shown in Figure 1D, the contact angle of PLGA layer was 147.5 ± 2.96° while the Gel layer was 21.2 ± 1.36°, which demonstrated that the asymmetric wettable membrane was successfully prepared. Figure S3 showed the water droplet could be diffused on the surface of hydrophilic Gel layer, but the spherical shape can be maintained on the surface of hydrophobic PLGA layer, which further revealed the excellent asymmetric wettability of the membrane.

### 3.2 Physical properties of the asymmetric membrane

The ideal wound dressings should have a suitable SR, which will help to absorb exudates of wound site and keep moisture environment on wound bed.^12^ As shown in Figure 1E, the SR values during the first 2 h for Gel, PLGA, and Gel/PLGA were 16.50, 2.27, and 4.02, respectively. Then the SR will remain stable and basically unchanged for 24 h. The high SR was mainly due to the high hydrophilicity (such as hydroxyl, carboxyl, and amino groups) of Gel and porous structure of membrane.^31^ However, the low SR for the Gel/PLGA membrane was related to strong hydrophobicity of the PLGA.^32^

WVTR is a critical parameter of the dressing, which can maintain moisture microenvironment during wound healing.^33^ As shown in Figure 1F, the measured WVTR values for Gel, PLGA, and Gel/PLGA were 3112, 1129, and 2252 g/m^2^·24 h, respectively. In contrast to the other, the high WVTR value for Gel was due to the large pore size of Gel membrane. However, the low SR for PLGA membrane was related to its hydrophobic surface. Prior study reported that the wound dressings with WVTR range about 2000–2500 g/m^2^·24 h could keep moisture environment, which could contribute to promoting proliferation of epidermal cells and fibroblasts.^30^ All results indicated that prepared asymmetric membrane was close to this range and will help to promote wound healing and prevent the scars.

As shown in Figure 1G, the tensile strength of Gel/PLGA membrane was 2.29 ± 0.06 MPa, which is significantly higher than the Gel membrane (0.75 ± 0.05 MPa). The low tensile strength of Gel was due to the intermolecular hydrogen bonds in gelatin chains. Meanwhile, the high tensile strength of Gel/PLGA is related to the PLGA, and the tensile strength of the pure PLGA layer is 5.28 ± 0.06 MPa. As shown in Figure 1H, Young's modulus was used to evaluate the strength and stiffness of materials. Young's modulus of the Gel, PLGA, and Gel/PLGA were 159.63 ± 12.36, 78.56 ± 4.90, and 117.38 ± 10.06 MPa, respectively. The low Young's modulus of PLGA was due to the low crosslinking density which could be observed in the above SEM image.^34^ Our results showed that the Gel/PLGA with appropriate mechanical properties will be suitable for wound healing applications.

**FIGURE 2:**
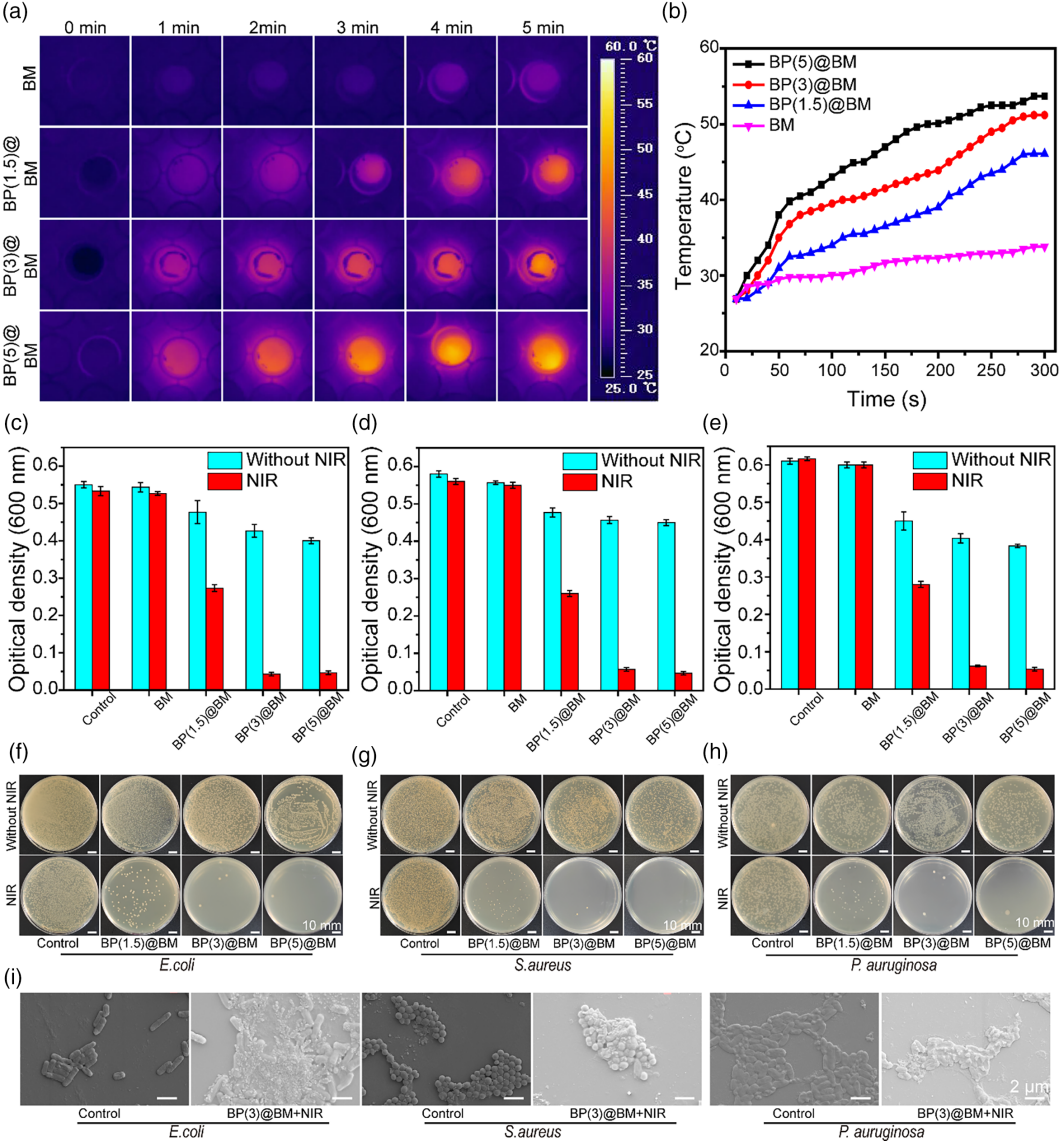
Physical properties of the asymmetric membrane. (A) Infrared thermal images of membrane irradiated using NIR laser (808 nm, 1.5 W/cm^2^). (B) Photothermal heating curves of the membrane with different concentration of BP irradiated by NIR laser (808 nm, 1.5 W/cm^2^). Viability of *Escherichia coli* (C), *Staphylococcus aureus* (D), and *Pseudomonas aeruginosa* (E) treated with different materials. Images of agar plates of *E. coli* (F), *S. aureus* (G), and *P. aeruginosa* (H) treated with different materials. (I) SEM images of *E. coli*, *S. aureus*, and *P. aeruginosa* treated with control and BP (3.0) @BM + NIR groups

The biodegradation of biomaterials was a critical factor in tissue engineering applications.^35^ As shown in Figure 1I, the degradation rate of Gel was 71.6% at 10 days, which was because the collagenase could decompose the gelatin chain. The degradation rate of PLGA was 99.2%, which exhibited almost no degradation. However, the Gel/PLGA degraded slowly and the degradation rate was 89.9%, which could offer the supportive environment that facilitated cell migration and adhesion during early wound healing process. The degradation rate of Gel, PLGA, and Gel/PLGA at 28 days were 48.8%, 97.6%, and 70.3%, respectively. Notably, the materials could not degrade completely within 28 days, which was due to that the collagenase degradation model in vitro could not simulate entirely the complex enzymatic environment *in vivo*.^36^ The standard curve of Rg1 is shown in Figure S4. As the results presented in Figure 1J, the Rg1/BP@BM membrane could promote the release Rg1 under the NIR excitation. Infrared thermal images exhibited the temperature at six time points (0, 1, 2, 3, 4, and 5 min) (Figure 2A). As shown in Figure 2B, the curve represented the change of temperature at different concentration of BP. The BP (5.0) @BM and the BP (3.0) @BM rapidly reached 50°C within 5 min. However, the BP (1.5) @BM attained 40°C after irradiation about 200 s and bellowed 50°C within 5 min. Prior study reported that bacteria could be damaged at the temperature of 50°C.^37^ Considering the high temperature may damage the skin, the group of the BP (3.0) @BM was selected to be the suitable concentration. Moreover, the BP (3.0) @BM could maintain the photothermal capacity after five on/off laser cycles at 5 min per cycle (Figure S5), which further indicated that the BP (3.0) @BM was able to serve as a potential biomaterial.

### 3.3 Antibacterial activity in vitro

Photo thermotherapy exhibited a broad spectrum of antibacterial effect, and was not prone to produce drug resistance. The antimicrobial efficacy of asymmetric wettable composite membrane was evaluated by *E. coli*, *P. aeruginosa*, and *S. aureus* with and without NIR irradiation. As presented in Figure 2C, Figure 2D, and Figure 2E, the BM group showed no antibacterial effect with or without NIR irradiation. Meanwhile, the BP (1.5) @BM group showed the limited antibacterial effect because the temperature was too low to restrain the bacteria. However, the BP (5.0) @BM and BP (3.0) @BM groups showed excellent antibacterial effect, which was owing to the fact that BP could convert light energy into high enough temperature under the NIR irradiation, resulting in the devastating destruction of bacteria.^23^ Notably, the BP (5.0) @BM and BP (3.0) @BM groups exhibited moderate antibacterial activity without NIR irradiation, which was related to the sharp edges, resulting in the damage of the bacterial membrane, which is similar to graphene and MoS_2._ ^38^ The images of agar plates with *E. coli* (Figure 2F), *P. aeruginosa* (Figure 2G), and *S. aureus* (Figure 2H) showed direct antibacterial activity, which was consistent with the above results. As shown in Figure 2I, *E. coli*, *S. aureus*, and *P. aeruginosa* of the control groups showed smooth and intact membrane. However, the bacteria of the BP (3.0) @BM + NIR group showed an abundant presence of cracks as well as bacterial shrink and collapse. Figure S6 shows the hydrophobic surface of BM showing bacterial antiadhesion ability, which caused the weak cell affinity because of a lack of cell recognition sites, further inhibiting the adhesion of bacteria.^39^

### 3.4 Cytotoxicity, cell migration, and angiogenesis in vitro

**FIGURE 3:**
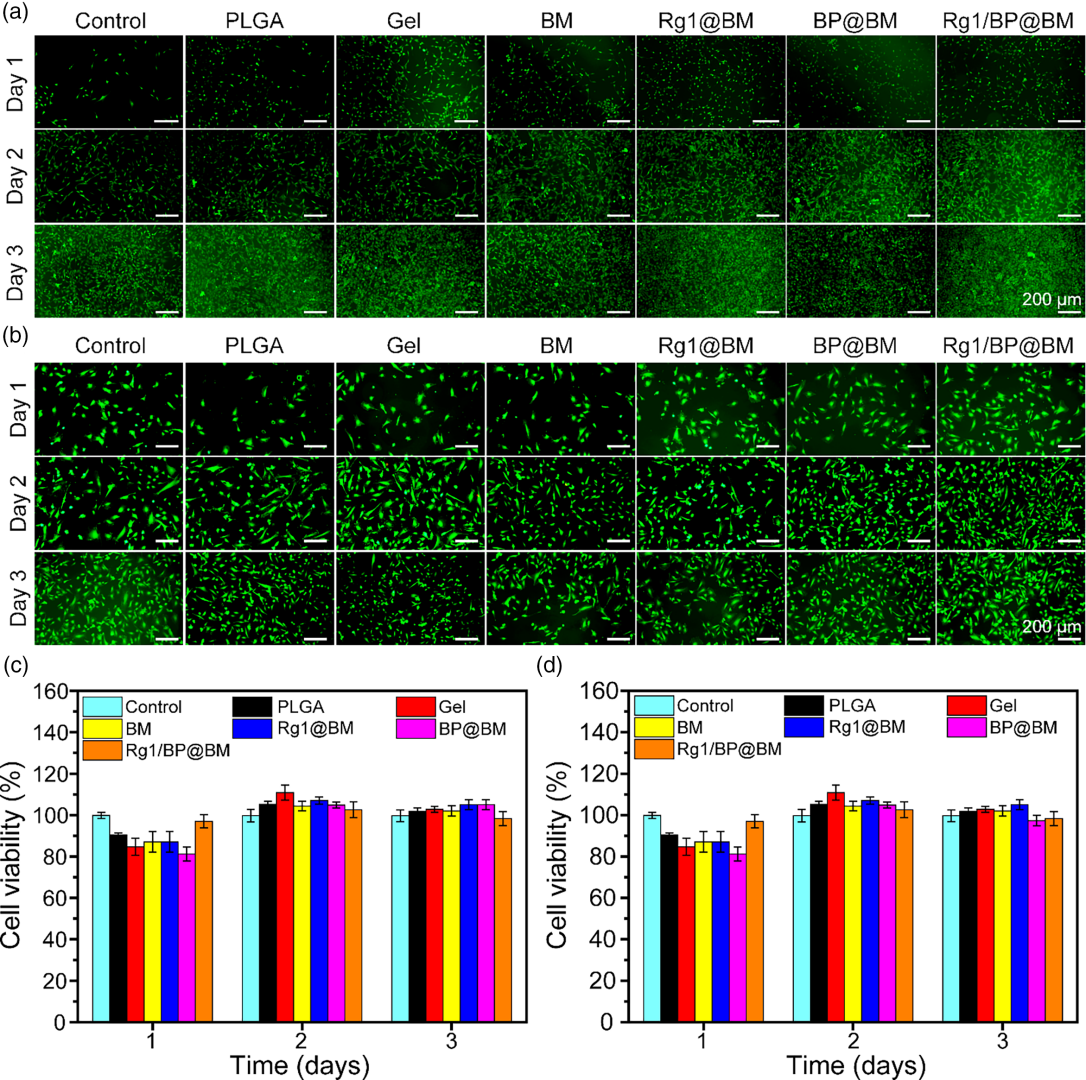
Biocompatibility evaluation of asymmetric membrane. Live/dead staining images of 3T3 cells (A) and HUVECs (B) at days 1, 2, and 3. Cell viability 3T3 cells (A) and HUVECs (B) versus different culture times by CCK‐8 assay

The biocompatibility of membranes was evaluated by live‐dead staining and CCK‐8 assay. Figure 3A,B showed the live‐dead staining of 3T3 and HUVECs cells, respectively. Most of the 3T3 and HUVECs cells were alive after co‐culturing with different membranes. Moreover, the cell viability was consistent with the staining results (Figure 3C and Figure 3D). The cell viability of 3T3 in Rg1/BP@BM group was 97.0%, 102.6%, and 98.3% after culturing day 1, day 2, and day 3, respectively. Meanwhile, the cell viability of HUVECs was 97.0%, 102.6%, and 98.3%, respectively. There was no significant difference of cell viability between the Rg1/BP@BM and the control. The Rg1/BP@BM showed slightly lower viability, which was due to that the degradation products of BP are phosphates and phosphonates, which are of low toxicity to cells and harmless to humans.^24^ All results revealed that all membranes exhibited no distinct toxicity to 3T3 and HUVECs, which were similar to the previous study.^40^ The Rg1/BP@BM with superior biocompatibility has potential prospect in the biomaterials field.

**FIGURE 4:**
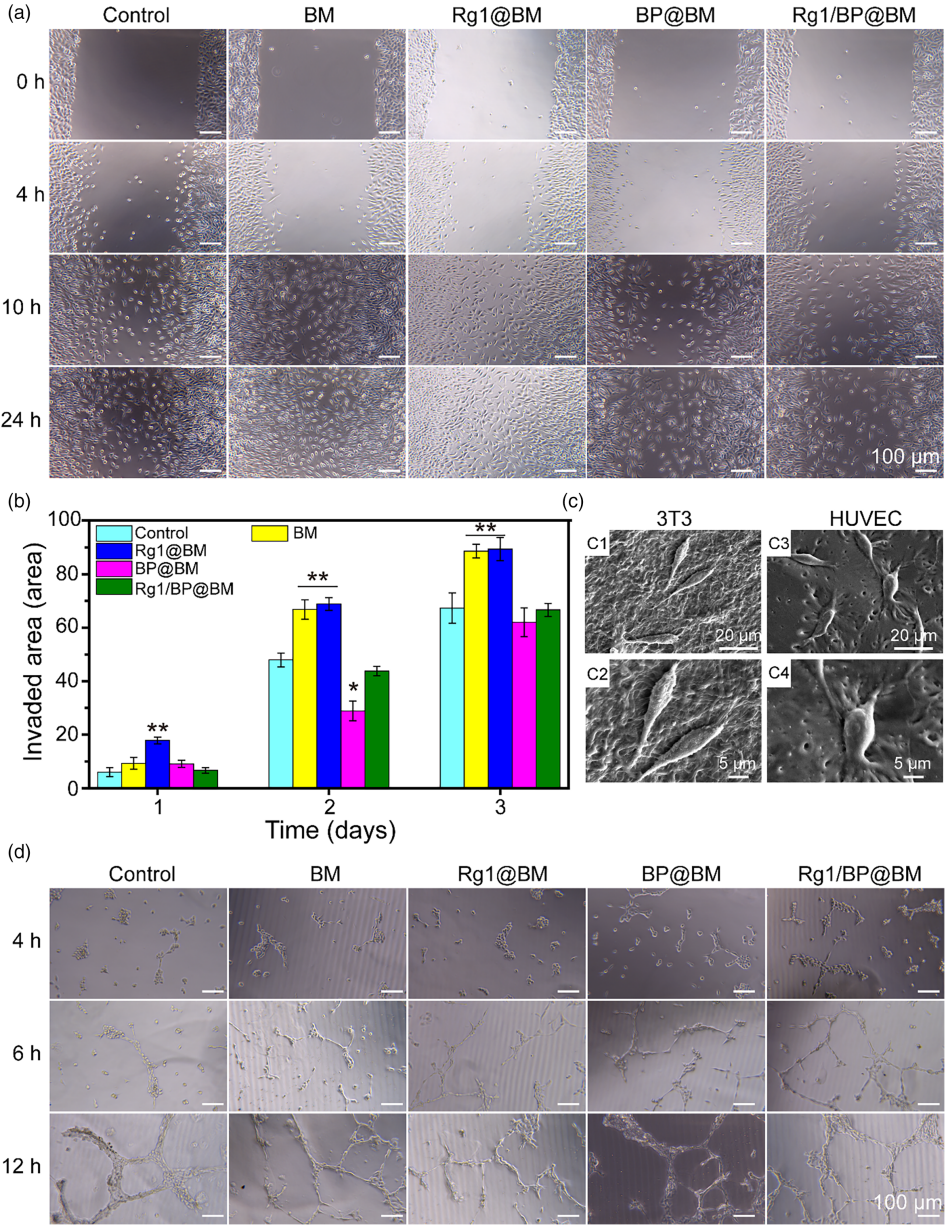
In vitro wound healing assay of asymmetric membrane. (A) The optical images of the in vitro wound healing experiment of HUVECs. (B) Quantitative invaded area. (C) The growth state of 3T3 and HUVECs on the electrospinning membrane. (D) In vitro tube formation of HUVECs cultured with different materials

The influence of the different membranes on HUVECs migration was determined using scratch assay. The photographs of scratch and the percentage of wound closure are shown in Figure 4A, B. At 0 h, the scratches with the same width were fabricated at bottom of each well. At 24 h, Rg1 containing Rg1@BM and Rg1/BP@BM groups presented faster migration of HUVECs and more superior wound closure than the control group. Moreover, the statistical analysis of the invaded area exhibited the similar results, which indicated that Rg1 could promote fibroblast cell migration. The SEM images revealed that the 3T3 and HUVECs successfully and tightly adhered to the electrospinning membrane and exhibited same linear cell morphology with normal cultured cells (Figure 4C). The pro‐angiogenic ability of HUVECs from different membranes was evaluated by tube formation. As shown in Figure 4D, the HUVECs of all groups formed capillary reticular structure after 6 h. Compared with the control group, the Rg1@BM and Rg1/BP@BM groups significantly increased the tube length and the number of nodes after 24 h, which was due to the fact that Rg1 could promote angiogenesis.^25^, ^41^

### 3.5 In vivo wound healing evaluation in rat wound infection model

**FIGURE 5:**
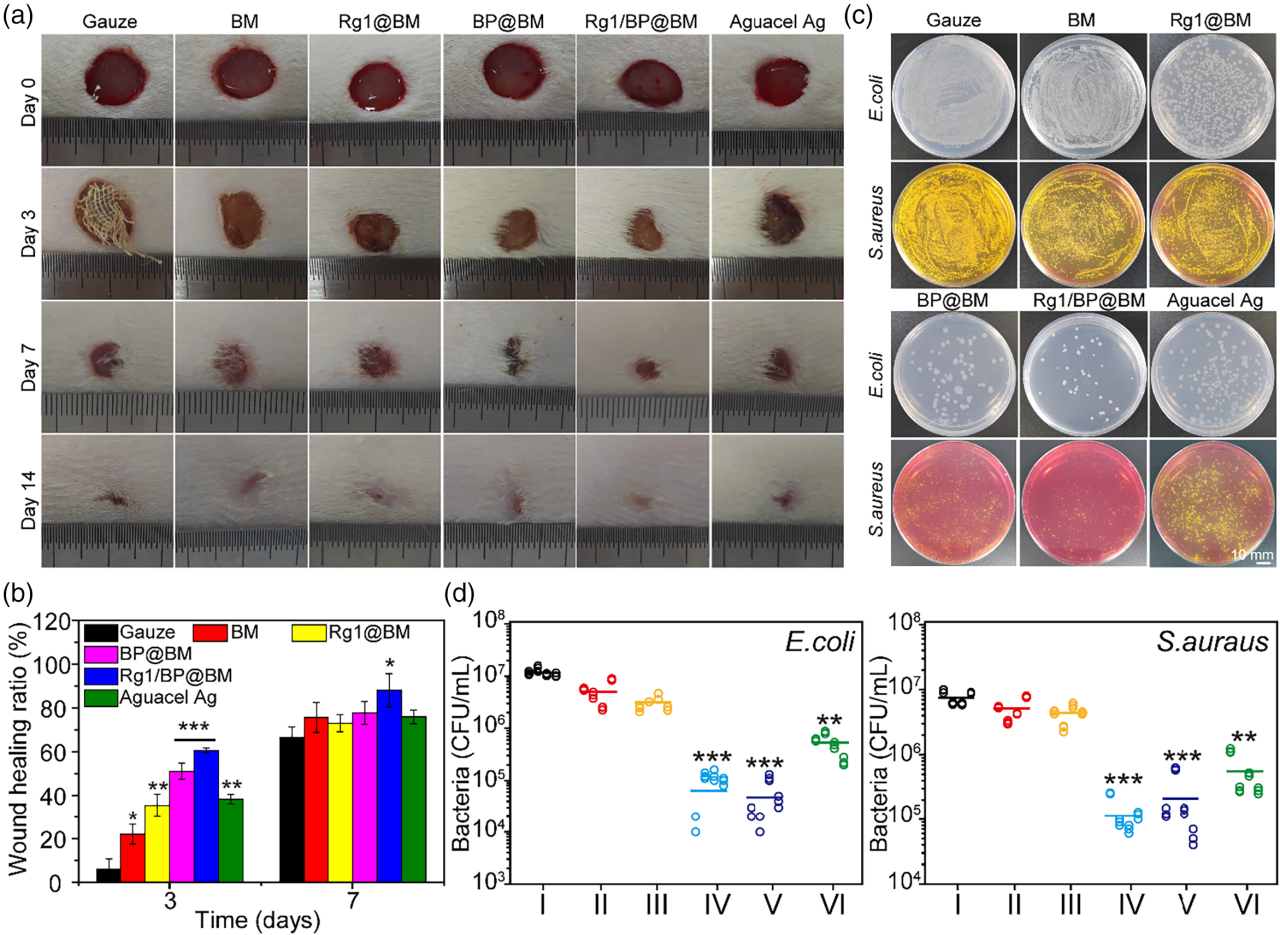
In vivo evaluation of membrane for wound healing. (A) Photographs of wounds treated by the different materials on days 0, 3, 7, and 14. (B) Percent change in wound size at different healing times. (C) *Staphylococcus aureus* and *Escherichia coli* colonies remaining in the wound area at day 3. (D) Numbers of viable *S. aureus* and *E. coli*

To verify the effects of prepared asymmetric membrane on wound healing, in vivo wound closure activity of asymmetric membrane in rat wound model with bacterial infection was evaluated. At 7‐ and 14‐day post‐operation, the wound area exhibited the obvious differences. As shown in Figure 5A, the Rg1/BP@BM presented the better therapeutic effect than other groups on the 3rd day and 7th day, and the wound has been completely healed on the 14th day. Besides, the statistical analysis (Figure 5B) of wound area further proved the difference of the groups which correspond to the situation of the wound images. Furthermore, the number of bacteria as shown in Figure 5C, D, which further demonstrated that the Rg1/BP@BM has the excellent antibacterial activity in vivo.

**FIGURE 6:**
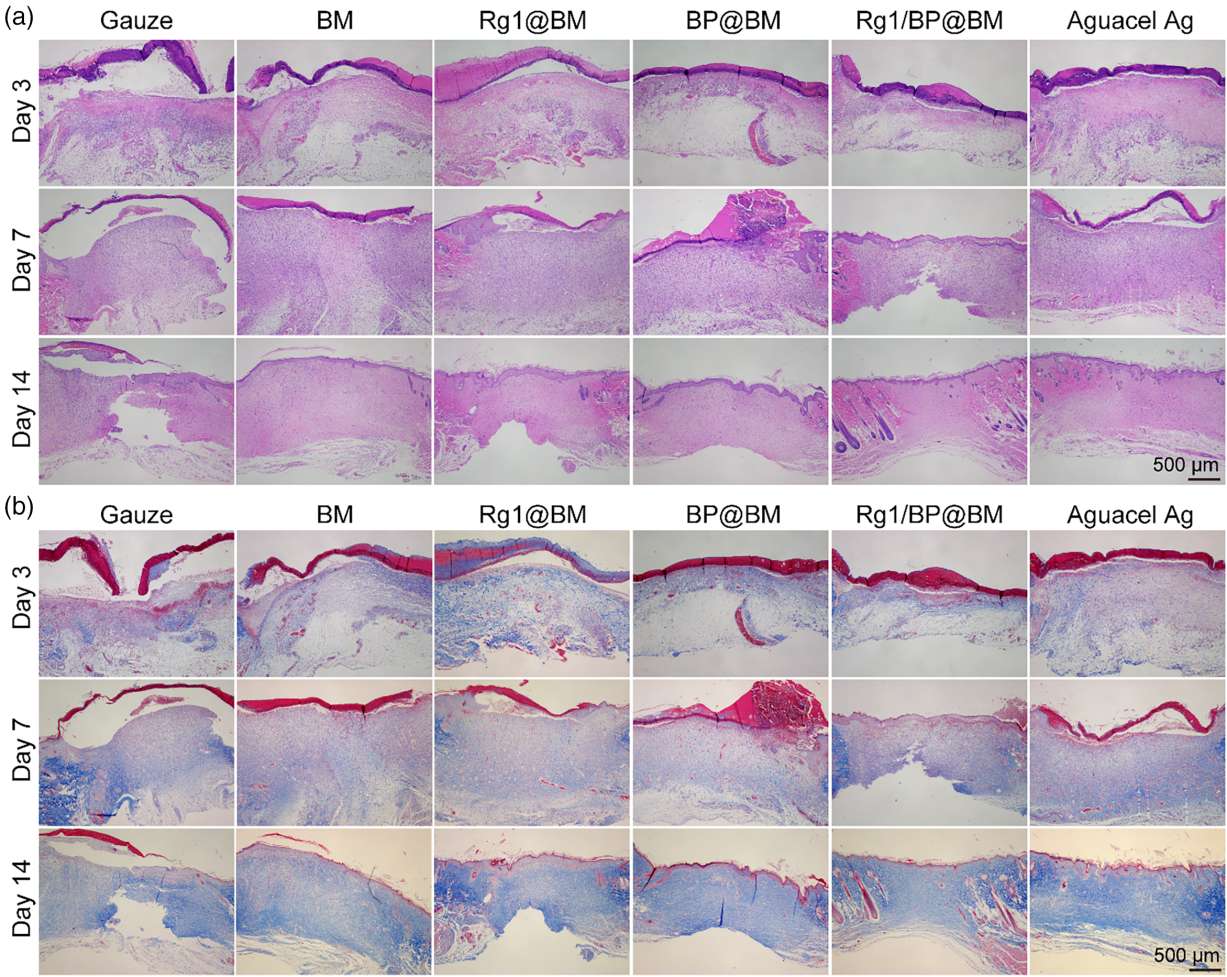
(A) H&E staining of wound tissues. (B) Masson's trichrome (MT) staining of wound tissues

The H&E staining of skin tissues was used to analyze the effects of membrane. As shown in Figure 6A, the necrotic tissue and a vast inflammatory cell of all groups could be observed in the epidermis on the 3rd day. However, the gauze group could observe the numerous inflammatory cells than other groups on the 7th day. Meanwhile, the Rg1/BP@BM group could observe the regenerated epidermis. On the 14th day, all the groups observed the regenerated epidermis tissue. The tissue separation between the epidermis and dermis could be observed in the gauze group. However, the Rg1/BP@BM and Aquacel Ag appeared at the regenerated accessory skin organs like papillary layer and sebaceous glands, which indicated that the structure of epidermis and dermis have been remodeled completely. Collagen fiber was stained blue with Masson's trichrome (MT), which was used to assess the collagen deposition.^42^ As shown in Figure 6B, the collagen of all groups was gradually increased during the wound healing. On the 3rd day, the collagen deposition of all groups exhibited no significant difference in the dermis. However, the collagen fiber in Rg1/BP@BM showed basket‐weave organization on the 7th day, and other groups were still low and irregular (Figure S7). On the 14th day, the group treated with Rg1/BP@BM exhibited a large amount of collagen deposition, and the collagen fiber was regular and thick as normal tissue.

### 3.6 The mechanism of wound healing

**FIGURE 7:**
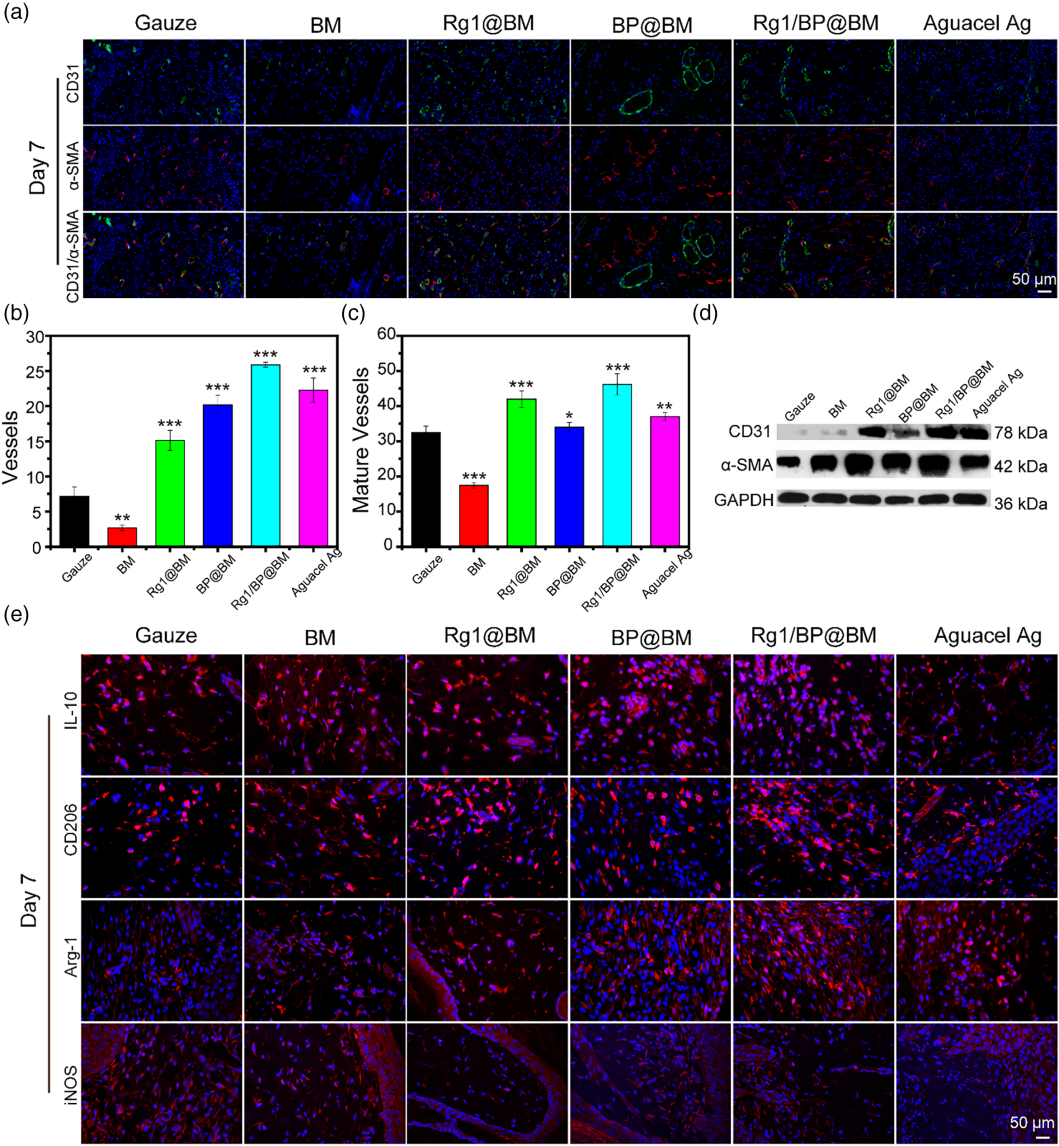
(A) Immunofluorescence staining of CD31 and α‐SMA of wound sections at day 7. Quantitative analysis newly formed blood vessels (B) and mature blood vessels (C) at day 7. (D) Western bolt analysis of CD31 and α‐SMA. (E) Representative IL‐10, CD206, Arg‐1, and iNOS images of IF staining of wound sections at day 7. Scale bar: 50 μm

Angiogenesis was a crucial mechanism in transportation of oxygen and nutrients to the wound area and new tissue.^43^ Besides, CD31 was a marker used to identify vascular endothelial cells, and α‐smooth muscle actin (α‐SMA) was a marker of the actin isoform typically used to identify smooth muscle cells.^44^ Vascular endothelial cells could be stained by CD31 to red fluorescence, and the green fluorescence represented the formation of blood vessels that were stained by α‐SMA. As shown in the Figure 7A–C, the Rg1/BP@BM presented more mature blood vessels than other groups at 7 days post‐operation, which was due to that Rg1 could promote vascularization according to previous study.^25^ WB of CD31 and α‐SMA further supported immunohistochemistry results (Figure 7D). Previous study reported that the wound healing is a complex and multi‐faceted biological process, which can be schematically separated into four phases: hemostasis, inflammation, proliferation, and remodeling.^45^ Classical M1 macrophages could generate pro‐inflammatory mediators to resist infection, while M2 macrophages promoted disappearance of inflammation, and enhanced the tissue remodeling.^46^ The immunofluorescent analyses of M1 maker (iNOS) and M2 maker (IL‐10, CD206 and Arg‐1) were used to estimate the polarization state. As shown in Figure 7E, the Rg1/BP@BM exhibited higher expression of IL‐10, CD206, and Arg‐1 and lower expression of iNOS. The ratio of fluorescence intensity of iNOS/Arg‐1 to measure the dynamic regulation of M1/M2 polarization of macrophages by different materials at the 7th day. Figure S8 showed that the Rg1/BP@BM has the lowest value of M1/M2, which indicated that Rg1/BP@BM promoted M2 polarization of macrophages and inhibited M1 polarization of macrophages, resulting in promoting the wound healing.

**FIGURE 8:**
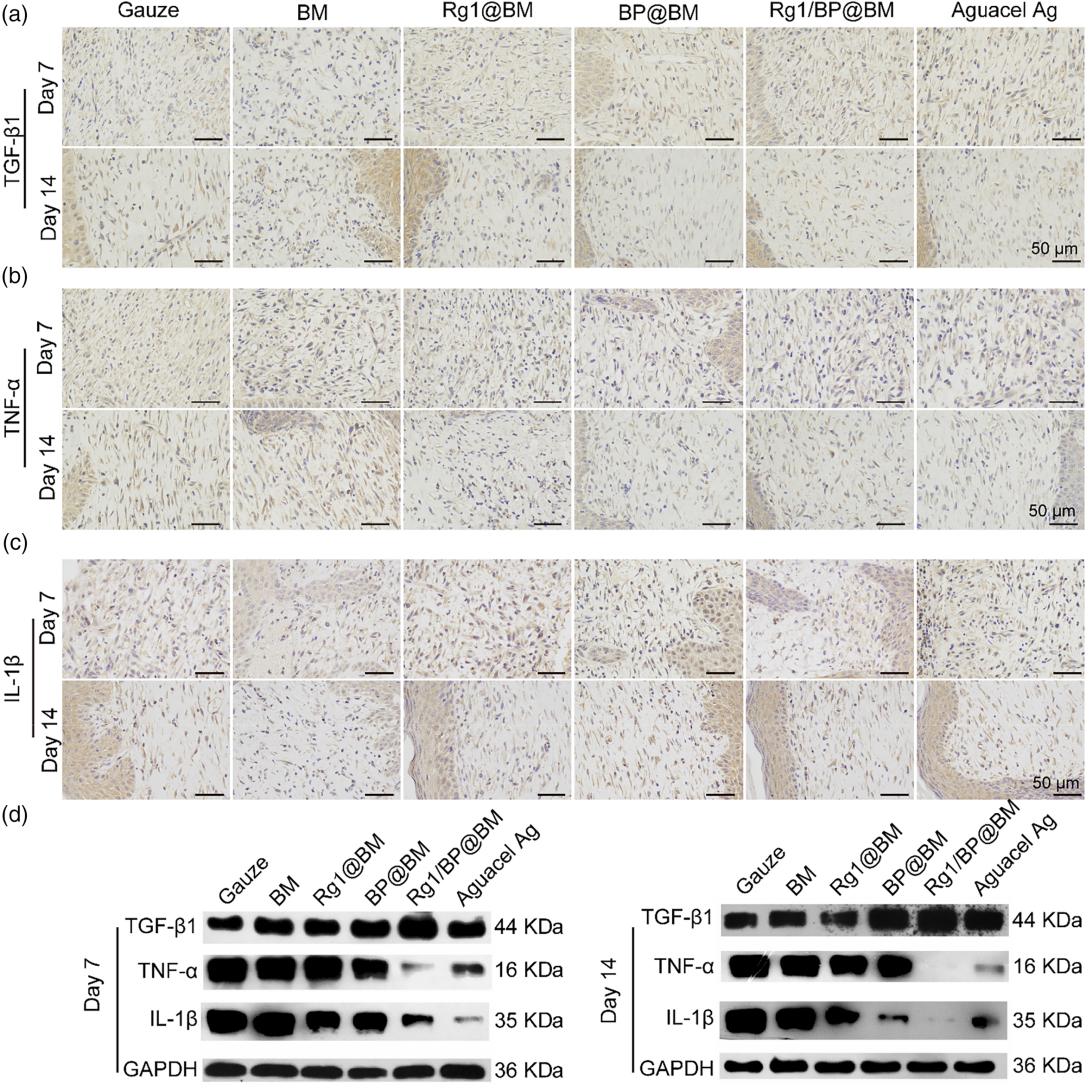
Representative images of TGF‐β1 (A), TNF‐α (B), and IL‐1β (C) expression at wounds by Immunohistochemistry staining at day 7 and day 14. (D) WB analysis of TGF‐β1, TNF‐α, and IL‐1β at day 7 and day 14. Scale bar: 50 μm

The expression of several pro‐inflammatory cytokines could be induced by inflammatory cells and played the important role in defense responses in early wound healing process.^47^ TGF‐β1 is the anti‐inflammatory cytokine that could promote wound healing. As shown in Figure 8A, the BP@BM, Rg1/BP@BM, and Aquacel Ag groups exhibited high expression of TGF‐β1 at the 7th day, while the gauze, BM, Rg1@BM group showed low expression at 14th day. TNF‐α and IL‐1β were the typical pro‐inflammatory factors that could assess the anti‐inflammatory effects during wound healing process. As shown in Figure 8B, C, the BP@BM, Rg1/BP@BM, and Aquacel Ag groups could down‐regulate expression of TNF‐α than the other groups at the 7th day. However, the gauze, BM, Rg1@BM groups showed higher expression of TNF‐α at 14 days post‐operation. The expression of the IL‐1β in different groups was similar to TNF‐α. WB analysis of TGF‐β1, TNF‐α, and IL‐1β exhibited a similar trend in the regulation of inflammation (Figure 8D). The Rg1/BP@BM and Aquacel Ag groups showed low expression of TGF‐β1, TNF‐α, and IL‐1β, while the gauze, BM, Rg1@BM, BP@BM groups exhibited high expression both at 7‐days and 14‐days post‐operation, which indicated that Rg1/BP@BM could regulate the inflammation. All results proved that the prepared Rg1/BP@BM membrane could regulate the inflammation and enhance the collagen deposition.

### 3.7 WB analysis and signal pathway

**FIGURE 9:**
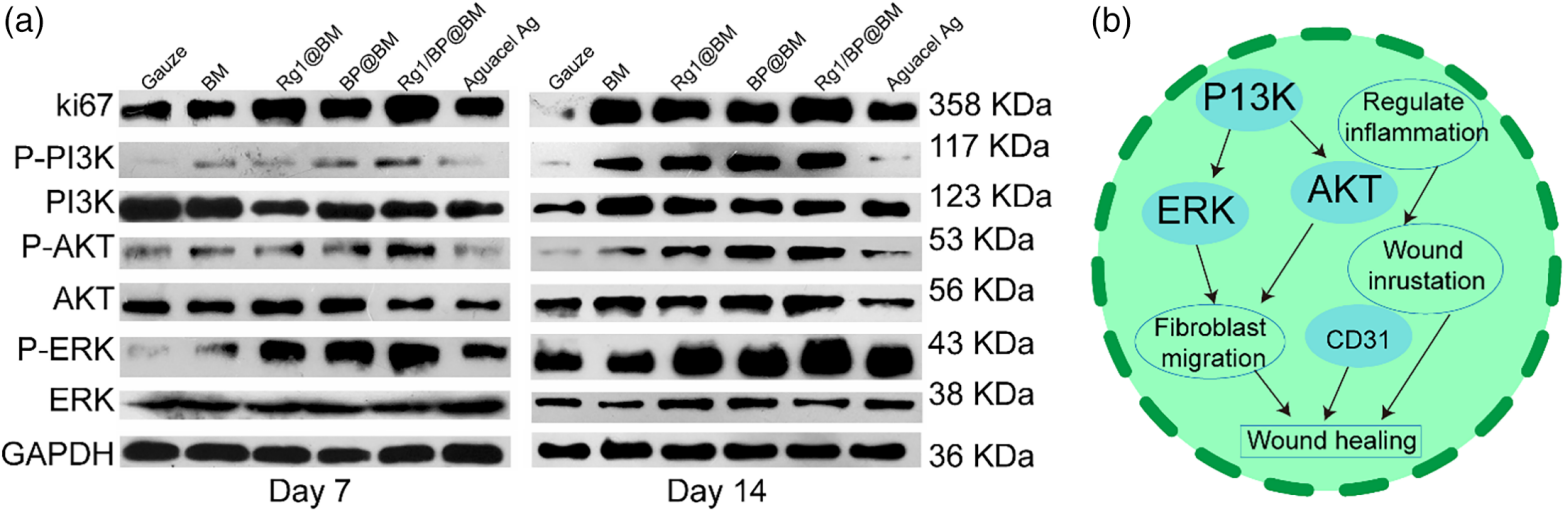
(A) WB analysis of ki67/P‐PI3K/PI3K/P‐AKT/AKT/PERK/ERK. (B) Signal pathway of the wound healing

WB analysis was used to test the protein expression level of ki67, P‐PI3K/PI3K, P‐AKT/AKT, and PERK/ERK. The signal pathway of ki67, PI3K/PI3K, P‐AKT/AKT, and PERK/ERK is a typical anti‐apoptosis pathway, which could regulate the promotion of cell growth, proliferation, and differentiation.^48^ As shown in Figure 9A, the Rg1/BP@BM showed higher protein expression than other groups, which indicated that it could effectively enhance cell growth, proliferation, and differentiation during wound healing process. In conclusion, the asymmetric wettable Rg1/BP@BM membrane could enhance the wound healing through regulating the signal pathway by inhibiting inflammation and promoting cell/fibroblast proliferation, collagen deposition, and angiogenesis (Figure 9B).

## 4 CONCLUSIONS

In summary, we successfully developed a symmetric wettable membrane for the treatment of chronic wound infections. The developed membrane had good swelling and mechanical properties, suitable WVTR and degradation, which could maintain the appropriate microenvironment for wound healing and cell proliferation. In vitro results revealed that the Rg1/BP@BM membrane could promote cell migration and proliferation and angiogenesis. In addition, the membrane with predominant photothermal performance and hydrophobic surface made it with excellent antibacterial activity, which could inhibit the adhesion of bacteria in vivo and in vitro. In the rat wound infection model in vivo, prepared Rg1/BP@BM membrane could accelerate wound healing processes through increasing expression level of anti‐inflammatory factors (TGF‐β1), reducing the expression of pro‐inflammatory factors (IL‐1β and TNF‐α), promoting M2 polarization (IL‐10, CD206 and Arg‐1) of macrophages, inhibiting M1 polarization (iNOS) of macrophages, promoting angiogenesis (CD31 and α‐SMA) at the molecular level, and regulating the signal pathway (ki67, PI3K/PI3K, P‐AKT/AKT, and PERK/ERK) at the protein level, all of which accelerate wound healing process via modulating the healing function of macrophage polarization, cell migration, angiogenesis, and tissue remodeling. More importantly, the proposed method by combining electrospinning with 2D material and traditional Chinese medicine may open new horizons in the design of new wound healing biomaterials.

## CONFLICT OF INTEREST

The authors declare no competing financial interest.

## AUTHOR CONTRIBUTIONS

**Nanbo Liu:** Conceptualization (equal); methodology (equal). **Longbao Feng:** Data curation (equal); resources (equal). **Mingyi Zhao:** Data curation (equal); investigation (equal); methodology (equal); software (equal). **Peng Wu:** Formal analysis (equal); software (equal). **Yunfei Chai:** Formal analysis (equal); software (equal).

### PEER REVIEW

The peer review history for this article is available at https://publons.com/publon/10.1002/btm2.10274.

## Supporting information


**Appendix** S1: Supporting Information

## Data Availability

Data available on request due to privacy/ethical restrictions
