# Supplementary material for: Multifunctional electrospun asymmetric wettable membrane containing black phosphorus/Rg1 for enhancing infected wound healing
Source: Bioeng Transl Med. 2021 Dec 15;7(2):e10274. doi: 10.1002/btm2.10274 (PMC9115714; doi:10.1002/btm2.10274)
Supplement: Supplementary file 1 — Appendix S1: Supporting Information [file BTM2-7-e10274-s001.docx]

**Supporting Information**

**Multifunctional electrospun asymmetric wettable membrane containing black phosphorus/Rg1 for enhancing infected wound healing**

Liming Zhou ^a, 1^, Nanbo Liu ^b, 1^, Longbao Feng ^a, 1^, Mingyi Zhao ^b^, Peng Wu ^b^, Yunfei Chai ^b^, Jian Liu ^b *^ Ping Zhu ^b *^ Rui Guo ^a *^

^a.^ Key Laboratory of Biomaterials of Guangdong Higher Education Institutes, Guangdong Provincial Engineering and Technological Research Centre for Drug Carrier Development, Department of Biomedical Engineering, Jinan University, Guangzhou, 510632, China

^b.^ Guangdong Cardiovascular Institute, Guangdong Provincial People's Hospital, Guangdong Academy of Medical Sciences, Guangzhou, Guangdong 510100, China

^1^ These authors contributed equally to this work.

^*^ Corresponding author: [guorui@jnu.edu.cn (Rui](mailto:guorui@jnu.edu.cn%20(Rui) Guo), [tanganqier@163.com](mailto:tanganqier@163.com) (Ping Zhu), jameslau1984@sina.com (Jian Liu)


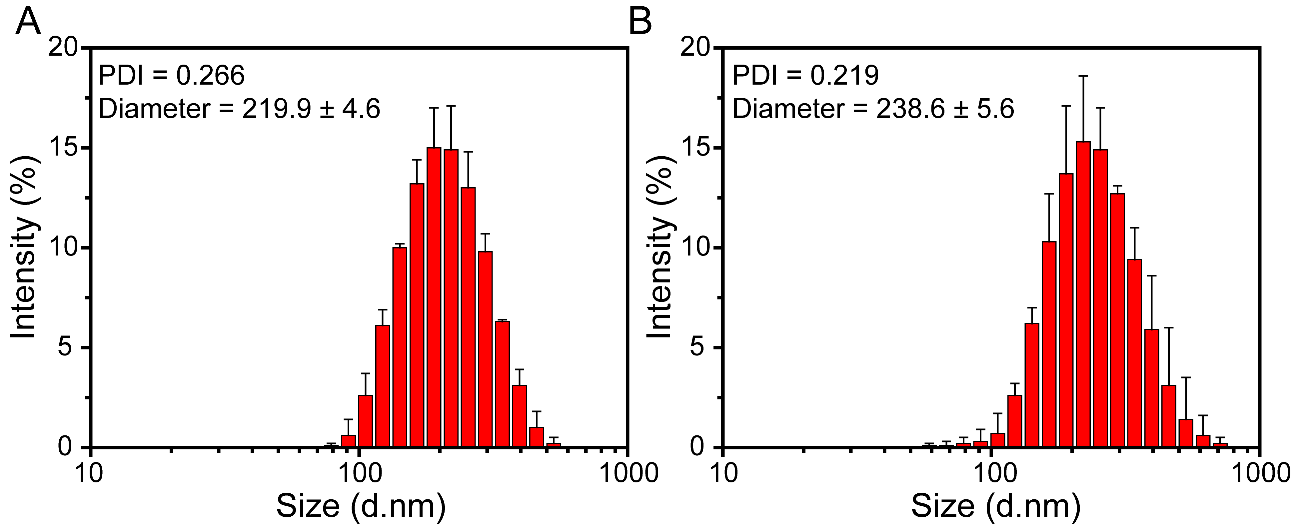


**Figure S1.** The diameter of the black phosphorus (BP) and black phosphorus grafts chitosan (HACC-BP).


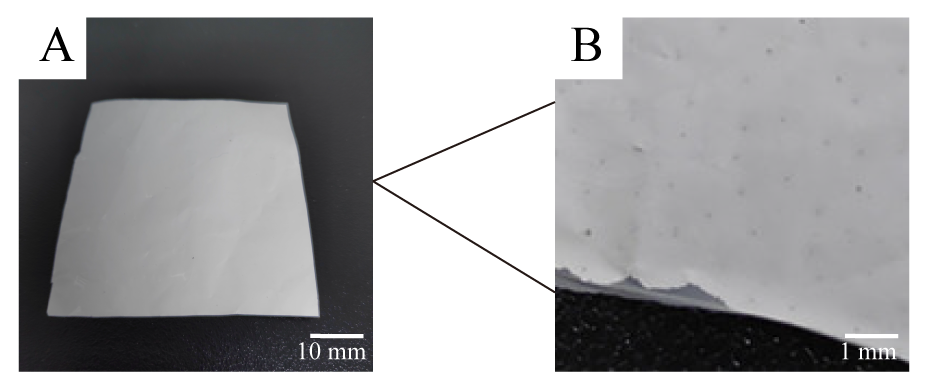


**Figure S2.** The optical images of the asymmetric membrane.


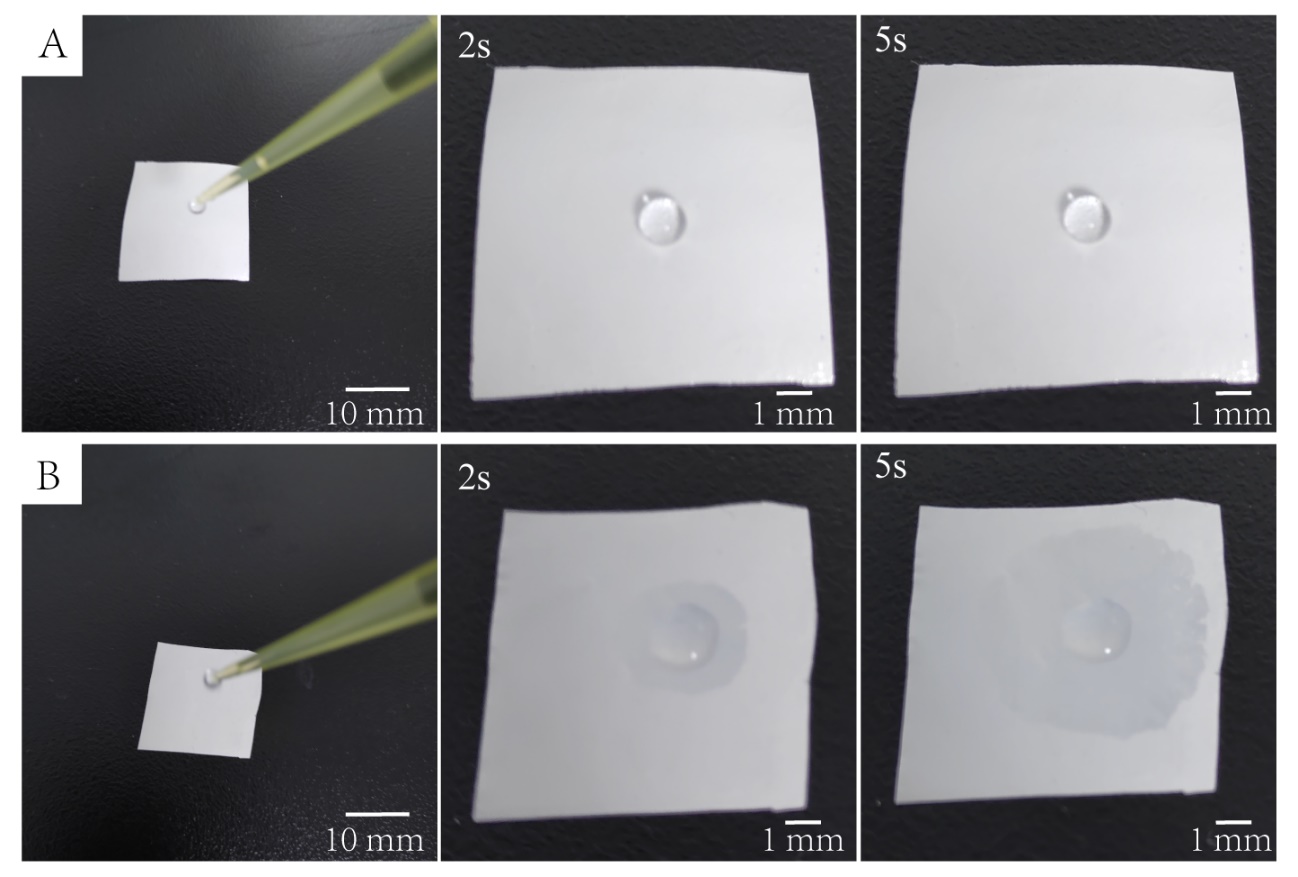


**Figure S3.** The water droplet on the hydrophilic layer surface (A) and the hydrophobic layer surface (B).





**Figure S4.** The standard curve of Rg1.





**Figure S5.** Temperature changes of BP (3.0) @BM groups with 808 nm laser switch-on or switch-off for five times.


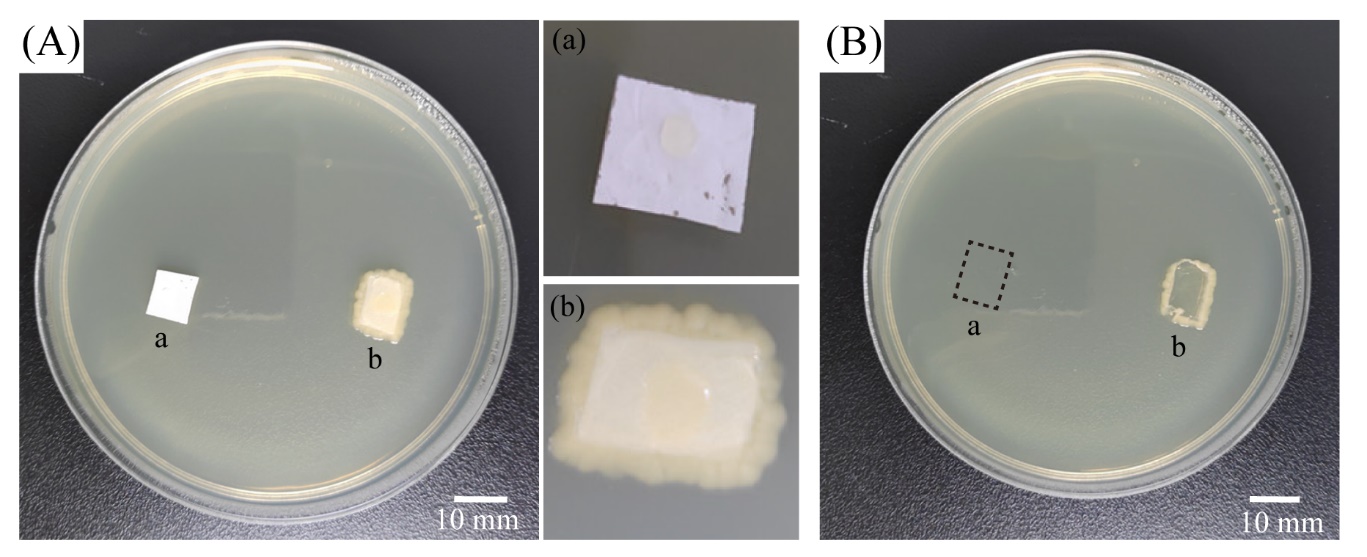


**Figure S6.** Antibacterial permeability picture of Gel (a) and Gel/PLGA (b) membrane.


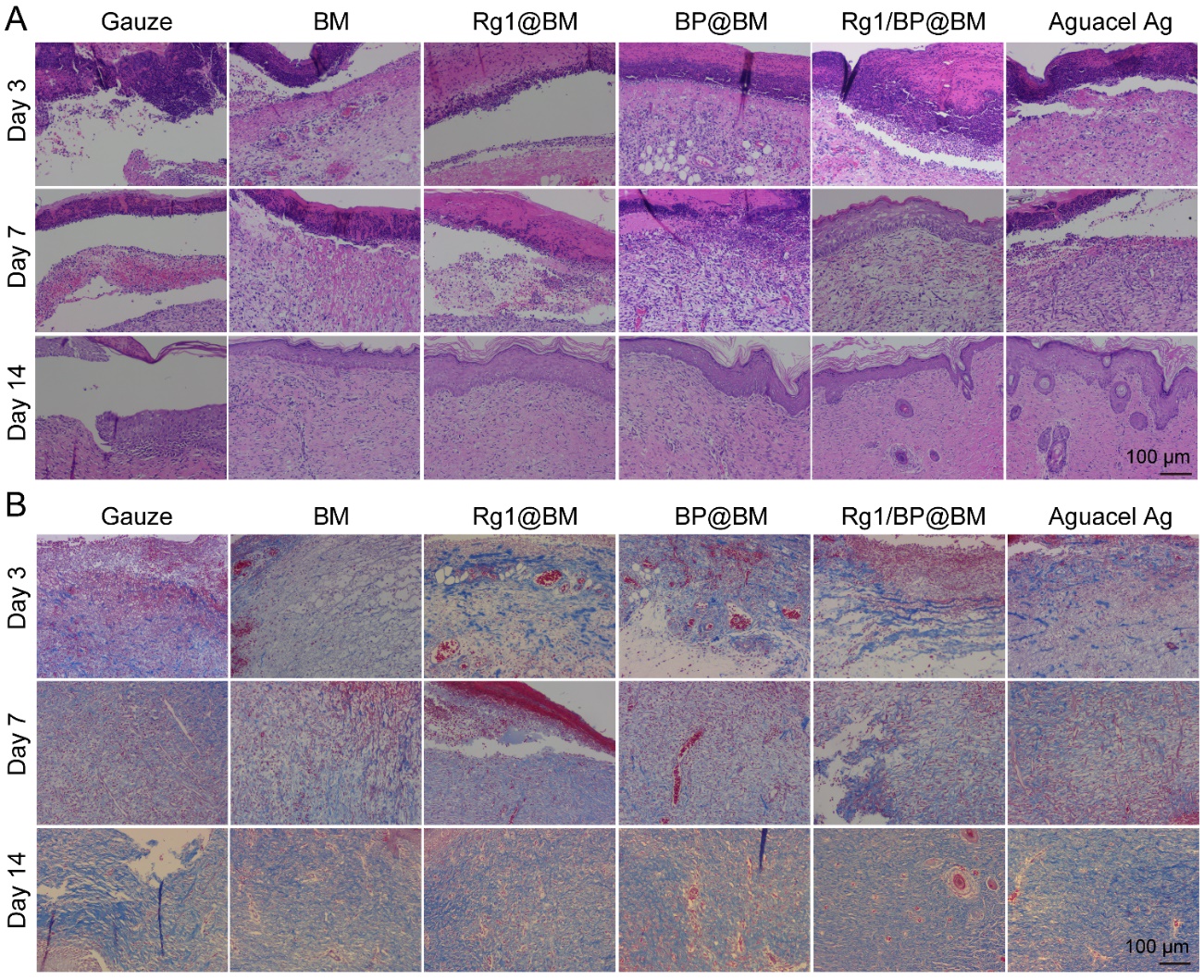


**Figure S7.** The local images of (A) H&E staining and (B) Masson’s trichrome staining of the wound section at days 3, 7, and 14. Scale bar: 100 μm.





**Figure S8.** Effects of different materials on M1/M2 ratio at the 7^th^ day.
